# Long-term clinical and angiographic outcome of the Woven EndoBridge (WEB) for endovascular treatment of intracranial aneurysms

**DOI:** 10.1038/s41598-022-14945-w

**Published:** 2022-07-06

**Authors:** Lukas Goertz, Thomas Liebig, Eberhard Siebert, Franziska Dorn, Muriel Pflaeging, Robert Forbrig, Lenhard Pennig, Marc Schlamann, Christoph Kabbasch

**Affiliations:** 1 Department of Radiology and Neuroradiology, Faculty of Medicine and University Hospital, University of Cologne, Kerpener Strasse 62, 50937 Cologne, Germany; 2 Department of Neuroradiology, University Hospital Munich (LMU), Marchioninistrasse 15, 81377 Munich, Germany; 3 Department of Neuroradiology, University Hospital of Berlin (Charité), Charitéplatz 1, 10118 Berlin, Germany; 4 Department of Neuroradiology, University Hospital of Bonn, Venusberg-Campus 1, 53127 Bonn, Germany

**Keywords:** Cerebrovascular disorders, Brain

## Abstract

The Woven EndoBridge (WEB) is a well-established device for endovascular treatment of wide-necked bifurcation aneurysms. The objective was to evaluate the long-term angiographic outcome of the WEB and to identify factors that influence aneurysm occlusion. Patient, aneurysm and procedural characteristics of 213 consecutive patients treated with the WEB at three German tertiary care centers between 2011 and 2020 were retrospectively reviewed. Aneurysm occlusion was determined immediately after the procedure, at mid-term (≤ 12 months) and at long-term (> 12 months) follow-up. Among 182 included aneurysms (mean diameter: 7.0 ± 2.4, mean neck width: 4.3 ± 1.6 mm), 29.7% were ruptured. The novel WEB 17 was used in 41.8%, and 11.0% were treated in combination with coiling and/or stenting. Complete and adequate occlusions were observed in 101/155 (65.2%) and 133/155 (85.8%) at mid-term, respectively, and in 59/94 (62.8%) and 87/94 (92.6%) at long-term follow-up (median: 19 months), respectively. Among 92 patients available for both mid- and long-term follow-up, occlusion was stable in 72.8%, improved in 16.3% and worsened in 10.9%. There were no major recurrences leading to aneurysm remnants between mid- and long-term follow-up. Retreatment was performed in 10/155 (6.5%) during mid-term and in 1/94 (1.0%) during long-term follow-up. The WEB provides durable aneurysm occlusion at the long-term. Nevertheless, follow-up imaging is necessary to identify late recurrences that may occur in around 10%.

## Introduction

Intrasaccular flow-disruption with the Woven Endobridge (WEB; Sequent Medical, Aliso Viejo, CA, USA) is an innovative technique for endovascular treatment of wide-necked bifurcation aneurysms. After its introduction in 2011, several prospective good clinical practice studies and numerous retrospective case series demonstrated a favorable safety and efficacy profile of the WEB^2,9,21–23^. As a result, the WEB received FDA (Food and Drug Administration) approval in 2018 for the treatment of wide-necked aneurysms located at the anterior communicating artery (Acom), the middle cerebral artery (MCA) bifurcation, the internal carotid artery (ICA) terminus, and the basilar tip^1^. In particular, successful angiographic occlusion seems to exceed that of conventional coiling and is at least on a par with stent-assisted coiling for a similar subset of aneurysms^14,15,21^. In a recent meta-analysis, Zhang et al. reported complete occlusion in 53%, neck remnants in 27% and aneurysm remnants in 20% at a mean of 9 months after WEB treatment^31^. Whereas the mid-term angiographic results have been reported extensively, studies on long-term angiographic outcome are rare. In particular, the long-term fate and the clinical significance of small neck remnants, which can be observed frequently after WEB implantation due to slight shape modification at the device recess remains unclear^11^.

The objective of this retrospective, multi-center study was to evaluate mid- and long-term angiographic outcome of WEB embolization in consecutive patients. Moreover, we aimed to identify patient, procedural and aneurysm characteristics that influence aneurysm occlusion at the long-term.

## Results

### Patient and aneurysm characteristics

During the study period, a total of 213 patients were treated with the WEB for 214 aneurysms. After exclusion of 8 aneurysms with failed WEB implantation, 13 aneurysms > 11 mm, 9 previously treated, recurrent aneurysms and 2 partially thrombosed aneurysms, the final study population consisted of 181 patients with 182 aneurysms. The mean patient age was 58.3 ± 11.9 years and 129 patients (71.3%) were female. Among 58 patients (32.0%) with subarachnoid haemorrhage, the WEB was used for 54 ruptured (29.7%) and 4 secondary, unruptured aneurysms (2.3%). The aneurysms were located in the anterior circulation in 112 cases (60.1%) and in the posterior circulation in 70 (37.9%). The average aneurysm size was 7.0 ± 2.4 mm and the mean neck width was 4.3 ± 1.6 mm. Bifurcation location was seen in 145 aneurysms (79.7%) and 167 (91.8%) were wide-necked. Aneurysm characteristics are detailed in Table 1.

**Table 1:** Baseline aneurysm characteristics.

Parameter	Value (n = 182)
Ruptured aneurysm status	54 (29.7%)
Lobular shape	16 (18.8%)
**Aneurysm location**	
Acom	49 (26.9%)
Pericallosal	3 (1.6%)
MCA	28 (15.4%)
**ICA**	
Terminus	4 (2.2%)
Paraophthalmic	13 (7.7%)
Pcom	15 (8.2%)
BA	62 (34.1%)
VA	2 (1.1%)
SUCA	2 (1.1%)
PICA	4 (2.2%)
Bifurcation location	145 (79.7%)
**Aneurysm size**	
Dome width (mm)	6.1 ± 2.1
Height (mm)	6.5 ± 2.5
Maximum diameter (mm)	7.0 ± 2.4
Neck width (mm)	4.3 ± 1.6
D/N ratio	1.5 ± 0.5
Aspect ratio	1.6 ± 0.8
Wide neck	167 (91.8%)

*Acom* anterior communicating artery, *PA* pericallosal artery, *MCA* middle cerebral artery, *ICA* internal carotid artery, *Pcom* posterior communicating artery, *BA* basilar artery, *VA* vertebral artery, *SUCA* superior cerebellar artery, *PICA* posterior inferior cerebellar artery, *D/N ratio* dome-to-neck ratio.

### Procedural details

Among 182 aneurysms, 162 were treated with WEB only (89.0%) and 20 (11.0%) in combination with other techniques (coiling: 3, stents: 15, stent-assisted coiling: 2). The WEB DL was used in 12 cases (6.6%), the WEB SL in 135 (74.2%) and the WEB SLS in 35 (19.2%). The WEB 17 was employed in 76 cases (41.8%). Immediately after WEB implantation, 89 of aneurysms (48.9%) were completely occluded, 33 (18.1%) had neck remnants, and 60 (33.0%) had aneurysm remnants (Table 2).

**Table 2:** Angiographic results immediately after WEB embolization, at mid-term follow-up (≤ 12 months) and at long-term follow-up (> 12 months).

	Immediate (N = 182)	Mid-term (N = 155)	Long-term (N = 94)
Follow-up period (months), median (IQR)	–	5 (1–7)	19 (15–26)
Complete occlusion	89 (48.9%)	101 (65.2%)	59 (62.8%)
Neck remnants	33 (18.1%)	32 (20.6%)	28 (29.8%)
Aneurysm remnants	60 (33.0%)	22 (14.2%)	7 (7.4%)
Recurrence	–	19 (12.3%)	10/92 (10.9%)
Major	–	8 (5.2%)	0
Minor	–	11 (7.1%)	10 (10.9%)
Stable occlusion	–	82 (52.9%)	67/92 (72.8%)
Progressive occlusion	–	54 (34.8%)	15/92 (16.3%)
Retreatment	–	10 (6.5%)	1 (1.0%)

*IQR* interquartile range.

### Mid-term angiographic follow-up

Mid-term angiographic follow-up was available for 155 aneurysms (85.2%) showing complete occlusion in 101 (65.2%), neck remnants in 32 (20.6%) and aneurysm remnants in 22 (14.2%). Hence, adequate occlusion was achieved in 85.8%. The recurrence rate was 12.3% (minor recurrence 7.1%, major recurrence 5.2%). Aneurysm occlusion was stable in 52.9% and progressive occlusion occurred in 34.8%. Ten patients (6.5%) with aneurysm remnants at mid-term follow-up were retreated (coiling: 2, stent: 4, stent-assisted coiling: 4) resulting in complete occlusion in 8 cases (80.0%) and a neck remnant in 2 cases (20.0%).

Patient age, gender, SAH, ruptured aneurysm status, aneurysm location, aneurysm size, neck width, size indices, aneurysm morphology, WEB type, treatment modality and immediate aneurysm occlusion were correlated with incomplete occlusion and recurrence at mid-term follow-up by univariate analyses. Factors associated with incomplete occlusion were: (1) Ruptured aneurysm status (vs. unruptured: 54.0% vs. 25.7%, p < 0.001), (2) a large aneurysm diameter (7.9 ± 2.6 mm vs. 6.8 ± 2.1 mm, p = 0.002), (3) a wide neck (4.8 ± 1.8 mm vs. 4.1 ± 1.4 mm, p = 0.007), and (4) immediate incomplete occlusion (vs. immediate complete occlusion: 51.8% vs. 16.2%, p < 0.001). A larger aneurysm height correlated with aneurysm recurrence (7.8 ± 2.5 mm vs. 6.4 ± 2.4 mm, p = 0.019).

### Long-term angiographic follow-up

Long-term angiographic follow-up was available for 94 aneurysms (51.6%) with a median follow-up period of 19 months (IQR 15–26). Complete occlusion was observed in 62.8%, neck remnants in 29.8% and aneurysm remnants in 7.4%. The adequate occlusion rate was 92.6%. Among 92 patients available for both mid- and long-term follow-up, there were 10 (10.9%) minor recurrences and no major recurrences. Reasons for aneurysm recurrence were WEB compression in 8 cases (80%) and WEB migration in disproportional high aneurysms in 2 cases (20%). There was not aneurysm growth. Sixty-seven (71.3%) aneurysms had stable occlusion and 15 (16.0%) had progressive occlusion. One patient with a neck remnant at mid-term follow-up was retreated by stent-assisted coiling (2.1%).

Univariate analyses were performed to correlate incomplete occlusion and recurrence between mid- and long-term follow-up with patient age, gender, SAH, ruptured aneurysm status, aneurysm location, aneurysm size, neck width, size indices, aneurysm morphology, WEB type, treatment modality and immediate/mid-term aneurysm occlusion. A large neck width was associated with long-term incomplete occlusion (4.8 ± 2.0 mm vs. 3.9 ± 1.1 mm, p = 0.01). No factors were significantly associated with delayed recurrence between mid-term and long-term follow-up.

### Complications and clinical outcome

Symptomatic complications occurred in 6 patients (3.3%), thereof, two were fatal. In the first patient, aneurysm perforation occurred during detachment of the WEB leading to fatal SAH. In the second patient, a separation thrombus occluded an M2 branch of the middle cerebral artery, which could not be resolved with tirofiban and resulted in ischemic stroke. Therapy was finally discontinued due to poor prognosis in the context of aneurysmal SAH. Furthermore, there were three minor infarcts and one perforating artery rupture which were associated with temporary neurological symptoms that resolved during follow-up. There were no WEB-related complications during follow-up. The overall morbidity rate was 3.3%. Favourable outcome was achieved in 98.3% at discharge and in 98.9% at final follow-up. There were no aneurysm (re-)bleedings or ischemic strokes during follow-up.

## Discussion

The key results of the present study were that adequate occlusion rates were 85.8% at the mid-term and 92.6% at the long-term follow-up. The recurrence rate between mid- and long-term follow-up was 10.9%, but there were no major recurrences leading to aneurysm remnants. Procedural morbidity was minimal with > 98% achieving favourable clinical outcome at follow-up.

Numerous studies have reported mid-term angiographic results of the WEB device, which are usually bound to a 6- or 12-month follow-up period^2,17,19,30^. In a recent meta-analysis by Zhang et al., the complete and adequate occlusion rates were 53% and 80% with a mean follow-up period of 9 months^31^. In the present study, mid-term complete and adequate occlusion rates were 69.9% and 89.5%, which were within the confidence intervals reported in the meta-analysis by Zhang et al.

Due to its spherical shape and its shape memory, the WEB was initially supposed to be less prone to compaction than coils. However, WEB shape modification occurs in around 30% which can appear as partial reperfusion of the aneurysm neck on angiography^11^. This phenomenon has also been denoted as “WEB compression” and describes a decrease in the device height due to the deepening of the device recess at the center^11^. These neck remnants are expected to be stable during long-term follow-up and do not generally require retreatment^5,11,29^. Hence, complete occlusion and neck remnants are often subsumed as “adequate occlusion” in many WEB studies^22,23,25^. Nevertheless, the clinical significance of neck remnants remains a matter of debate. Critics argue that neck remnants due to WEB compression may not be neglected because neck remnants carry an inherent risk for further worsening, rupture or re-rupture, as shown for coiling^4,6^. In the CARAT study, for instance, the risk of rehemorrhage after coiling of ruptured aneurysms was 1.1% for completely occluded aneurysms, 2.9% for small residual necks, 5.9% for neck remnants and 17.6% for aneurysm remnants^12^. Whether these data can be transferred to WEB treatment remains unclear due to a shortage of long-term data.

In the current study, long-term complete and adequate occlusion rates were 62.8% and 92.6%, respectively, which were comparable than those of mid-term follow-up. Minor recurrences occurred in 10.9% and there were no major recurrences between mid- and long-term follow-up.

There are only few studies on the long-term angiographic outcome after WEB implantation, their key results are summarized in Table 3. The largest study has been published by Pierot et al. reporting the 2-year results in the cumulative population of the three prospective WEBCAST (WEB Clinical Assessment of Intrasaccular Aneurysm Therapy), WEBCAST-2 and French Observatory trials^24^. Complete occlusion was observed in 51% and adequate occlusion in 81%. Comparable to our data, the authors reported stable occlusion in 80.7%, improved occlusion in 5.9% and recurrence in 13.4% between the 1-year and 2-year follow-up. The major recurrence rate was 3.4% beyond the 1-year follow-up and only these aneurysms would require retreatment in accordance with the current treatment recommendations. There were no aneurysmal bleedings or rebleedings. The authors also reported 3-year angiographic results of the WEBCAST and WEBCAST-2 trials, which were similar to the 2-year results^26^. The occlusion rates of these studies were low compared to the present study. This difference may be partially explained by core laboratory evaluation of aneurysm occlusion in the prospective trials, which typically leads to lower occlusion rates^28^. Moreover, the recruitment periods of the three prospective trials were between 2011 and 2015^24^. At that time, the technical experience with the WEB was limited and the methodology of WEB oversizing (+ 1/−1 rule) may not have been applied strictly in all participating centers^10^. Furthermore, these studies did not include newer-generation WEB types, which were associated with higher occlusion rates in the meta-analysis by Zhang et al.^31^. For comparison, the portion of aneurysms treated with the WEB 17 was 41.7% in the present study.

**Table 3:** Overview of studies reporting long-term outcome for the WEB.

Study	Follow-up period	Number of aneurysms	Complete occlusion	Neck remnant	Aneurysm remnant	Retreatment
Pierot et al.^24^	24 months (per protocol)	121	62 (51%)	36 (30%)	23 (19%)	11 (9%)
Fujimoto et al.^8^	6–24 months (mean: 15 months)	28	16 (57%)	5 (17%)	7 (25%)	6 (18%)
Mine et al. ^20^	24–72 months (mean: 25 months)	25	18 (72%)	7 (28%)	0 (0%)	4 (16%)
Present study	12–63 months (mean: 22 months)	94	59 (62.8%)	28 (29.8%)	7 (7.4%)	1 (1.1%)

To our knowledge, there are two further retrospective studies on the long-term angiographic outcome of the WEB: Mine et al. reported complete and adequate occlusion in 72% and 100%, respectively, which are slightly more favourable than our results^20^. In the study by Fujimoto et al. the complete and adequate occlusion rates were 57% and 74%, respectively^8^, which were comparable to the results reported in the prospective studies by Pierot et al.^24^.

The fate of residual aneurysm necks, which can be seen on mid-term follow-up angiography in approximately 30% after WEB implantation, is of particular interest for neurointerventionalists: Whereas aneurysm remnants represent a clear indication for retreatment, management recommendations for neck remnants are inconclusive and the physician has to decide between further follow-up or retreatment. Among 36 neck remnants at 1-year in the study by Pierot et al., 32 (89%) were stable or improved and 4 (11%) worsened to aneurysm remnants^24^. In the study by Mine et al., all 7 neck remnants at mid-term follow-up were stable at the long-term^20^. Likewise, in the present study, all neck remnants showed stable or improved occlusion at long-term follow-up. Adding up these numbers, among 75 neck remnants at mid-term follow-up reported in the literature, 71 (95%) were stable or improved at the long-term and 4 (5%) worsened. These data imply that neck remnants after WEB implantation do not require retreatment imperatively, however, further observation may be necessary in order to identify long-term recurrences. Due to a scarcity of data, we cannot give a definite management recommendation for neck remnants and we encourage other neurointerventionalists to share their experience.

In the present study, we identified several factors associated with incomplete aneurysm occlusion at mid- and long-term follow-up. Ruptured aneurysm status represents a well-known risk factor for recanalization after coiling^27^. In a previous paper by our group, occlusion rates were not significantly different between ruptured and unruptured aneurysms treated with the WEB^13^. Analyzing a larger patient collective in the present study revealed previous rupture to be an independent risk factor for incomplete aneurysm occlusion. In concordance, Zhang et al. reported lower occlusion rates for ruptured than for unruptured aneurysms^31^.

As previously demonstrated and also shown for coiling, a large aneurysm size and a wide neck were associated with incomplete aneurysm occlusion and recurrence^3^. In this context, Zhang et al. reported worse angiographic results for aneurysms with a very wide neck^31^. This observation may be explained by the fact that the effect of WEB compression becomes more pronounced with the use of large WEBs.

Similar to coiling, initial incomplete occlusion and a wide aneurysm neck correlated positively with incomplete occlusion at follow-up^18^. To minimize this effect, a slight oversizing of the WEB may be beneficial, as also suggested by other authors^10,23^.

In addition to our findings, Zhang et al. and König et al. reported improved angiographic results for the novel WEB 17^16,31^. This effect could not be demonstrated in our cohort and it remains unclear whether it is caused by the revised structure of the WEB 17, facilitated navigability and WEB positioning due to miniaturization or by consistent oversizing of the WEB in the recent years.

Finally, it has to be pointed out that these long-term results compare very favorably especially to conventional therapies for wide-necked bifurcation aneurysms including surgical clipping which are historically associated with relatively low rates of complete occlusion^7^.

## Limitations

The study is limited by its retrospective design and a moderate number of included patients. The aneurysms were heterogenous in size and configuration and different treatment methods were used for aneurysm embolization. Long-term angiographic outcome was limited and not available in all cases. Hence, patients with incomplete aneurysm occlusion at mid-term follow-up might have been more often subjected to long-term follow-up, which can bias the angiographic results. Moreover, WEB technique and devices evolved since its introduction in 2011, which could not be addressed in this study.

## Conclusions

The results of the current study demonstrate that WEB embolization provides durable aneurysm occlusion. Complete and adequate occlusion rates were 62.8% and 92.6% at long-term follow-up, respectively. Conceding that neck remnants are a frequent finding after WEB implantation, only 10.9% of aneurysm recurred between mid-term and long-term follow-up and there were no major recurrences. In particular, all neck remnants observed at the mid-term remained stable or improved at the long-term. Although WEB embolization provides long-term efficacy, follow-up imaging would be necessary to identify late recurrences in a small subset of aneurysms.

## Methods

The need for study approval for this retrospective, observational study was waived by the local ethics committees, the Ethics Commission of the Faculty of Medicine of Cologne University, the Ethics Commission of the Medical department of the Ludwig-Maximilians-University Munich and the Ethics Committee of Charité–Universitätsmedizin Berlin. Due to anonymized, retrospective data collection, the need for informed consent was waived by the local ethics committees (the Ethics Commission of the Faculty of Medicine of Cologne University, the Ethics Commission of the Medical department of the Ludwig-Maximilians-University Munich and the Ethics Committee of Charité–Universitätsmedizin Berlin). The study was conducted in accordance with the STROBE guidelines in compliance with the national legislation and the Code of Ethical Principles for Medical Research Involving Human Subjects of the World Medical Association (Declaration of Helsinki).

### Inclusion and exclusion criteria

This is a retrospective review of consecutive patients treated with the WEB at three German neurovascular centers between May 2011 and July 2020. The following aneurysms were included: (1) ruptured or unruptured aneurysm status, (2) treatment with all available WEB types (4) treatment with the WEB only or in combination with coiling and/or stent implantation. Exclusion criteria were: (1) WEB not implanted, (2) maximum aneurysm diameter $\ge$ 11 mm, (3) partially thrombosed aneurysms, and (4) previously treated, recurrent aneurysms.

### WEB treatment

The WEB procedure was performed via a transfemoral approach in a biplane angiosuite (Philips, Best, the Netherlands and Siemens, Erlangen, Germany). In most cases, the WEB was delivered through the dedicated VIA microcatheter (VIA 17, 21, 27 or 33, Sequent Medical, Aliso Viejo, CA, USA). In this study, the WEB single-layer (SL), double-layer (DL), and single-layer sphere (SLS) types were used. Since September 2020, all aneurysms with a maximum aneurysm diameter ≤ 7 mm were treated with the WEB 17 at all three institutions. Based on two-dimensional measurements on DSA images, the implant sizes were chosen to be slightly larger than the aneurysm equator diameter and slightly smaller than the aneurysm height (+ 1/−1 rule). Adjunctive devices were used on the discretion of the neurointerventionalist. In general, a stent was implanted to prevent WEB protrusion in aneurysms with unfavourable anatomy. If additional coiling was performed, we aimed to achieve complete aneurysm occlusion in aneurysms with atypical configuration.

### Anti-aggregation therapy

In elective cases, acetylsalicylic acid (ASA) 100 mg/day was administered for at least 4 weeks, starting 5–7 days before the procedure and a single bolus of heparin (5000 IU) was administered promptly after groin puncture, followed by aliquots of 1000 IU/hour during the intervention. In case of additional stent use, ASA 100 mg and clopidogrel 75 mg were administered for at least 4 months after the intervention. Thereafter, ASA monotherapy was continued. Patients with acutely ruptured aneurysms and need for additional intracranial stenting to optimize WEB positioning, received an intravenous infusion of tirofiban for 16–24 h, followed by a loading dose of ASA (150 mg) and clopidogrel (300 mg). Maintenance antiplatelet medication is similar to that of elective cases.

### Data collection

The following parameters were ascertained for this study: patient age, gender, subarachnoid haemorrhage, ruptured/unruptured aneurysm status, aneurysm location, WEB type, use of adjunctive stents or coiling and complications, morbidity and clinical outcome. Only symptomatic complications occurring during the hospital stay or during follow-up are reported and included predominantly ischemic and hemorrhagic events. Technical, asymptomatic events are not reported. Procedural morbidity was defined as a procedure-related increase of the modified Rankin scale (mRS) score ≥ 1 during the hospital stay or at follow-up. A mRS > 2 was defined as unfavourable outcome. Conventional four-vessel digital subtraction angiography (DSA) were reviewed to determine aneurysm dome width (D), height (H) and neck width (N) of the aneurysms. From these measurements, the maximum aneurysm diameter, the dome-to-neck (D/N) ratio and the aspect ratio (H/N) were calculated. A neck width ≥ 4 mm and/or a D/N ratio ≤ 2 were considered as wide-necked. Lobulated aneurysms were defined to have an additional aneurysm sac next to the main aneurysm sac.

### Angiographic control and retreatment

The standard follow-up protocol consisted of DSA, magnetic resonance angiography (MRA), or computed tomography angiography (CTA) at 6 and 24 months after the procedure. In case of incomplete aneurysm occlusion, follow-ups were scheduled on an individual basis.

Aneurysm occlusion was independently rated as complete occlusion, neck remnant, and aneurysm remnant by three experienced neurointerventionalists. Discrepancies were resolved in consensus. A slight filling of the device recess at follow-up was considered as complete occlusion. In order to facilitate comparison with previous WEB studies, complete occlusion and neck remnants were subsumed as adequate occlusion. The occlusion status of retreated aneurysm before retreatment was included in the long-term follow-up cohort, even if retreatment occurred within the first 12 months after treatment (mid-term follow-up). The morphological reasons for aneurysm recurrence (e.g. WEB compression, WEB migration, aneurysm regrowth) were evaluated.

A worsening of aneurysm occlusion at follow-up was defined as recurrence (neck remnant: minor recurrence, aneurysm remnant: major recurrence). Progressive aneurysm occlusion means improved aneurysm occlusion at follow-up. The decision towards retreatment or further observation of incompletely occluded aneurysms was made in consensus during an interdisciplinary neurovascular conference.

### Statistical analysis

Qualitative variables are presented as numbers and means and compared using the t-test and the Fisher Exact test. Quantitative variables are presented as means with standard deviation or as median with interquartile range (IQR) and compared using the Student’s t-test for normally distributed data and the Wilcoxon-Mann–Whitney-test for non-normally distributed data. Normality was assessed with the Shapiro–Wilk-test. Statistical analysis was performed using SPSS software (IBM SPSS Statistics for Windows, Version 25.0, Armonk, NY, USA). A p-value < 0.05 was considered as statistically significant.

### Informed consent

For this type of study formal consent is not required.

## Data Availability

All data will be made available upon request in an anonymized manner from the corresponding author LG (email: Lukas.goertz@uk-koeln.de).
